# Type I Interferons Direct Gammaherpesvirus Host Colonization

**DOI:** 10.1371/journal.ppat.1005654

**Published:** 2016-05-25

**Authors:** Cindy S. E. Tan, Clara Lawler, Janet S. May, Gabrielle T. Belz, Philip G. Stevenson

**Affiliations:** 1 School of Chemistry and Molecular Biosciences, University of Queensland and Royal Children’s Hospital, Brisbane, Australia; 2 Division of Virology, Department of Pathology, University of Cambridge, Cambridge, United Kingdom; 3 Molecular Immunology, Walter and Eliza Hall Institute, Parkville, Melbourne, Australia; University of Southern California, UNITED STATES

## Abstract

Gamma-herpesviruses colonise lymphocytes. Murid Herpesvirus-4 (MuHV-4) infects B cells via epithelial to myeloid to lymphoid transfer. This indirect route entails exposure to host defences, and type I interferons (IFN-I) limit infection while viral evasion promotes it. To understand how IFN-I and its evasion both control infection outcomes, we used Mx1-cre mice to tag floxed viral genomes in IFN-I responding cells. Epithelial-derived MuHV-4 showed low IFN-I exposure, and neither disrupting viral evasion nor blocking IFN-I signalling markedly affected acute viral replication in the lungs. Maximising IFN-I induction with poly(I:C) increased virus tagging in lung macrophages, but the tagged virus spread poorly. Lymphoid-derived MuHV-4 showed contrastingly high IFN-I exposure. This occurred mainly in B cells. IFN-I induction increased tagging without reducing viral loads; disrupting viral evasion caused marked attenuation; and blocking IFN-I signalling opened up new lytic spread between macrophages. Thus, the impact of IFN-I on viral replication was strongly cell type-dependent: epithelial infection induced little response; IFN-I largely suppressed macrophage infection; and viral evasion allowed passage through B cells despite IFN-I responses. As a result, IFN-I and its evasion promoted a switch in infection from acutely lytic in myeloid cells to chronically latent in B cells. Murine cytomegalovirus also showed a capacity to pass through IFN-I-responding cells, arguing that this is a core feature of herpesvirus host colonization.

## Author Summary

Gamma-herpesviruses establish chronic infections and cause cancers. They achieve this by immune evasion. Immune responses nonetheless suppress infection to a degree. By understanding how immune responses and viral evasion come together we can potentially control infection and prevent disease. MuHV-4 provides an accessible model with which to define outcomes. It enters new hosts via epithelial cells, passes to macrophages, then persists in B cells. Type I interferons—a key anti-viral defence—controlled epithelial and B cell infections but poorly restricted the intervening macrophage infection. Therefore for maximal effect interferons must act before B cells are infected.

## Introduction

The γ-herpesviruses persist in lymphocytes and cause lymphoid and epithelial cancers. MuHV-4, like Epstein-Barr virus (EBV) and the Kaposi's Sarcoma-associated Herpesvirus (KSHV), persists in B cells [1]. After epithelial entry, it reaches B cells in organized lymphoid tissue via dendritic cells (DC) [2]. It then spreads with remarkable precision from splenic marginal zone (MZ) macrophages to MZ B cells, follicular DC, then follicular B cells [3]. Glycoprotein conformation changes guide host colonization, with epithelial-derived virions infecting myeloid cells but not B cells, myeloid-derived virions infecting B cells, and B cell-derived virions infecting epithelial cells [4]. However epithelial-derived virions still infect epithelial cells better than myeloid cells, and myeloid-derived virions still infect epithelial and myeloid cells better than B cells. Therefore efficient B cell colonization must involve also a suppression of non-B cell infections.

Immune cell colonization makes host defences an important feature of the γ-herpesvirus infection landscape. Type 1 interferons (IFN-I) are a core vertebrate anti-viral defence [5]. Myriad stimuli induce IFN-I: classically double-stranded RNA (dsRNA), but also other nucleic acids in unusual forms or places, such as unmethylated and cytoplasmic DNA [6]. IFN-I secretion is triggered by phosphorylation of interferon regulatory factors (IRFs) 3 and 7. Signalling through the STAT-1/2-linked IFN-I receptor (IFNAR) then induces an anti-viral state in infected and surrounding cells via restricted protein synthesis, a reduced apoptosis threshold and immune effector recruitment. IFNα is produced mainly by myeloid cells, IFNβ by many cell types, and both in large amounts by plasmacytoid DC [7]. The multiplicity of induction pathways sensitive to different infection hallmarks ensures that essentially all viruses elicit an IFN-I response.

Most viruses also evade IFN-I [8]. MuHV-4 reduces IFN-I induction in infected cells, inhibiting IRF3 via ORF36 [9] and TANK binding kinase 1 via ORF11 [10]. It also reduces IFN-I signalling, down-regulating STAT-1 and STAT-2 via M2 [11], degrading IFNAR via ORF54 [12] and inhibiting responses downstream of IFNAR via ORF37 [13]. A third interaction is that IFN-I transcriptionally suppresses M2 to inhibit viral reactivation from latency, both directly [14] and by restoring STAT-1/2 expression to allow STAT-1-dependent transcriptional suppression of ORF50, the main viral lytic transactivator [15]. Such effects could explain why IFN-I retains an important role in preventing disease [16, 17]. However MuHV-4 still reactivates sufficiently to cause disease in IFN-I-competent mice lacking CD4^+^ T cells [18]; and M2 is still made sufficiently to promote acute lymphoid infection [19–21] and provide an important T cell target in long-term infection [22]. Thus, the quantitative relationships between IFN-I signalling and viral evasion remain unclear.

Qualitative questions also remain. ORF11, ORF36, ORF37, ORF54 and M2 all limit IFN-I signalling to infected cells, so ongoing viral gene expression should preclude a cellular IFN-I response; but in cells already exposed to IFN-I, viral signalling blocks should be ineffective and infection should be suppressed. In a uniform cell population such mutual inhibition would manifest as transient viral replication; but *in vivo* infection is more complicated, with MuHV-4 spreading between cell types and between anatomic sites [23]. Splenic B cell colonization involves at least 5 lytic cycles [3], so virus entry into IFN-I-exposed B cells must be common. For Herpes simplex virus, IFN-I does not prevent epithelial virus production but impaired responses promote pathological neuronal infection [24], suggesting that cell type is an important outcome determinant [25]. Most studies of MuHV-4 and IFN-I have averaged effects across whole organs, so how host response and viral evasion vary with cell type is unclear. To understand this better we tracked MuHV-4 replication in IFN-I responding cells. The results provide new insight into how a complex pathogen interacts with an ancient host defence, and how cell type-specific infection outcomes guide host colonization.

## Results

### IFN-I induction reduces MuHV-4 replication *in vitro*

**Fig 1:**
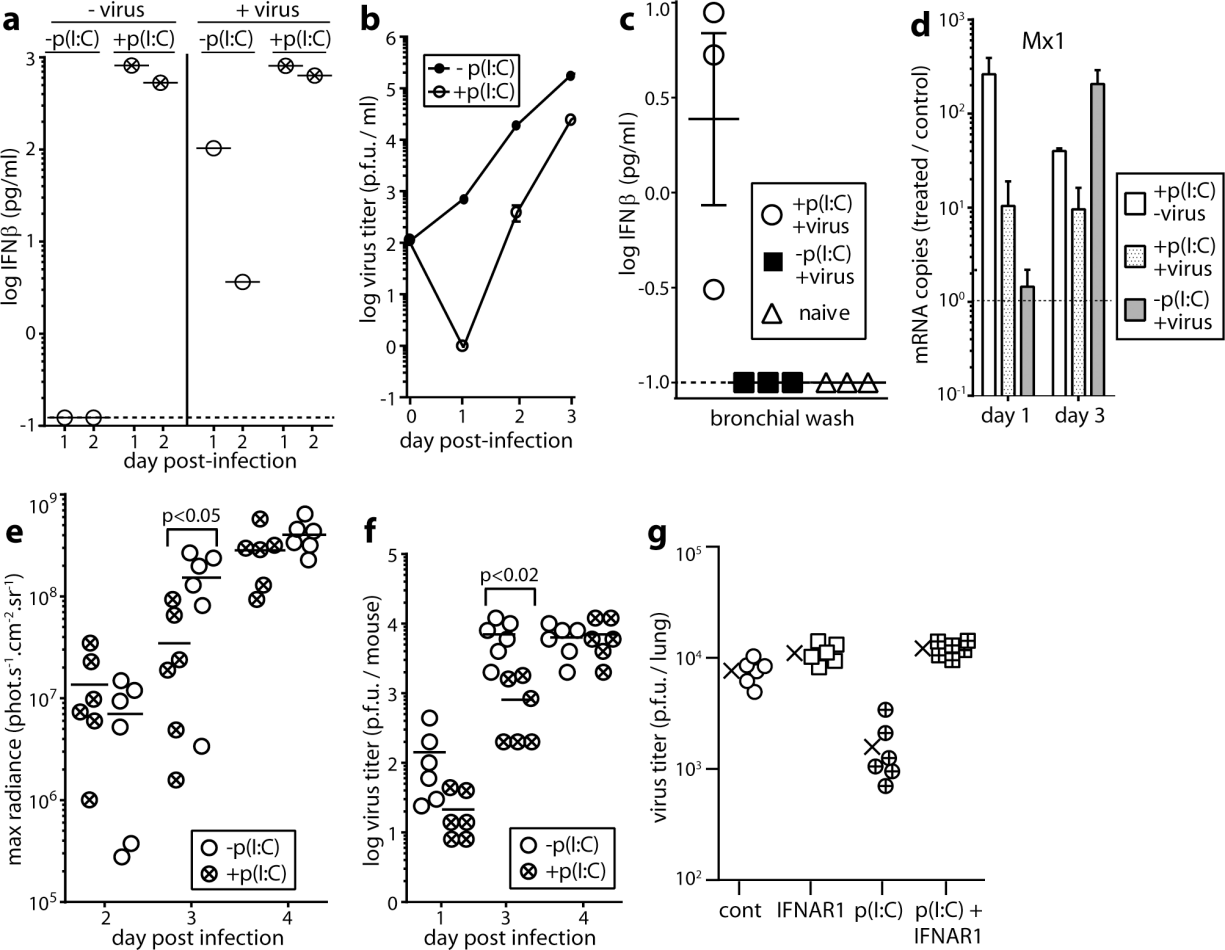
IFNαβ induction by and effect of IFNαβ induction on MuHV-4 infection *in vitro* and *in vivo*. **a.** RAW-264 monocyte/macrophages were infected or not with MuHV-4 (2 p.f.u. / cell) then cultured ± poly(I:C) as a positive control of IFN-I induction (50μg/ml). Cell supernatants pooled from triplicate cultures were assayed for IFNβ by ELISA 1 and 2d later. The dashed line shows the limit of assay sensitivity. A replicate experiment gave equivalent results. **b.** RAW264 cells were infected and cultured ± poly(I:C) to induce IFN-I as in **a**. Supernatants were assayed for infectious virus by plaque assay. Symbols show mean ± SEM of triplicate cultures. IFN-I induction significantly reduced virus titers from d1 onwards (p<0.03). **c.** Mice were given i.n. MuHV-4 (3x10^4^ p.f.u.), with or without poly(I:C) inoculation (50μg i.p. and i.n., 6h before and at the time of virus inoculation) as a positive control of IFN-I induction. After 1d bronchial washes were assayed for IFNβ by ELISA. Each point shows 1 mouse. Bars show mean ± SEM. The dashed line shows the limit of assay sensitivity. Poly(I:C) significantly increased IFNβ production in infected mice (p<0.01). **d.** Mice were infected i.n. or not with MuHV-4 with or without poly(I:C) treatment as in **c**. 1 and 3d later Mx1 mRNA in lungs was quantitated by real time PCR and normalized by cellular nidogen-1 copy number. Numbers show the fold increase in Mx1 copy number of treated mice over the untreated controls (dashed horizontal line). Bars show mean ± SEM of 3 mice. Mx1 copies were significantly increased by poly-IC at d1 (p<0.05) but not at d3. **e.** Mice were infected i.n. with MHV-LUC, and treated or not with poly(I:C) as in **c**. Lung infection was tracked by luciferin injection and live imaging of light emission. Circles show individual mice, bars show means. Poly(I:C) significantly reduced luciferase counts at d3 but not at d2 or d4. **f.** Mice were infected i.n. with MuHV-4 and treated or not with poly(I:C) to induce IFN-I as in **c**. Lungs were titered for infectious virus by plaque assay. Circles show individual mice, bars show means. Poly(I:C) significantly reduced infection at d3 but not at other time points. **g.** Mice were given an anti-IFNAR blocking antibody or not (200μg i.p.) 24h before and poly(I:C) or not (50μg i.p. and i.n.) 6h before i.n. infection with MuHV-4 (3x10^4^ p.f.u.). 3d later lungs were titered for infectious virus by plaque assay. Cont = virus only. Crosses show means, other symbols show individual mice. Poly(I:C) significantly reduced titers (p<0.01) and this effect was reversed by anti-IFNAR antibody.

MuHV-4 replicates in a wide range of primary and transformed cells, suggesting that it either induces little IFN-I or evades its effects. Weak inhibition of fibroblast infection by MuHV-4-expressed IFNα [26] suggests effector evasion. However the expression kinetics of IFNα were unclear, and the cells infected (hamster fibroblasts or IFNAR^-/-^ fibroblasts transfected with IFNAR encoding DNA) may have responded poorly to murine IFNα. Myeloid cells play a central role in MuHV-4 host colonization [27], and RAW-264 monocyte-macrophages are well-described IFN-I producers and responders [28]. Therefore we tested in RAW-264 cells whether MuHV-4 induced IFN-I, and whether IFN-I affected viral replication (Fig 1). ELISA of cell supernatants for IFNβ showed only limited induction in infected cultures (Fig 1A), so we tested viral susceptibility to IFN-I signalling more stringently by treating the RAW-264 cells with poly(I:C), a well-characterised TLR3 ligand. IFNβ was then readily detectable regardless of whether virus was present, and viral replication was reduced 10-fold (Fig 1B). However the infectivity of induced RAW-264 cell cultures still increased 100-fold between day(d)1 and d2 after MuHV-4 inoculation (Fig 1B), despite IFNβ being detected at both time points (Fig 1A), and continued to increase at d3. Therefore MuHV-4 both induced little IFNβ and partly resisted its effects.

### IFN-I induction transiently reduces MuHV-4 replication in lungs

We tested next how IFN-I affected MuHV-4 replication in the lungs, where it infects alveolar macrophages (AMs) and type 1 alveolar epithelial cells (AEC1s) [29]. Again we wanted to know whether IFN-I was induced, and if induced whether MuHV-4 resisted its effects. IFN-I induction is generally rapid, for example peaking 12-24h after experimental respiratory virus infection [30], but IFNAR^-/-^ mice given MuHV-4 i.n. have normal virus titers in the lungs for at least 4d [16], implying poor IFN-I induction or poor efficacy. Bioassays have suggested poor induction [31], and consistent with this result, IFNβ was not detected by ELISA of lung washes at 1d post-infection (Fig 1C). To test MuHV-4 resistance to IFN-I therefore, we induced it with poly(I:C) 6h before virus inoculation. This gave detectable IFNβ in lung washes (Fig 1C), and increased Mx1 transcription in lungs by d1, whereas infection took until d3 (Fig 1D). Live imaging of luciferase^+^ MuHV-4 (Fig 1E) and plaque assays of infectious virus (Fig 1F) showed IFN-I induction reducing lung infection at d3 but not at other time points. Although poly(I:C) also induces inflammatory cytokines, these seem not to affect acute MuHV-4 replication [32, 33] and IFNAR blockade abrogated the effect of poly(I:C) (Fig 1G). This implied also that IFN-III induction by poly(I:C) [34] did not have a marked effect. Thus, as with RAW-264 cell infection *in vitro*, MuHV-4 in the lungs induced little IFN-I, and inducing IFN-I only modestly reduced viral replication.

### MuHV-4 passes through IFN-I responding cells

**Fig 2:**
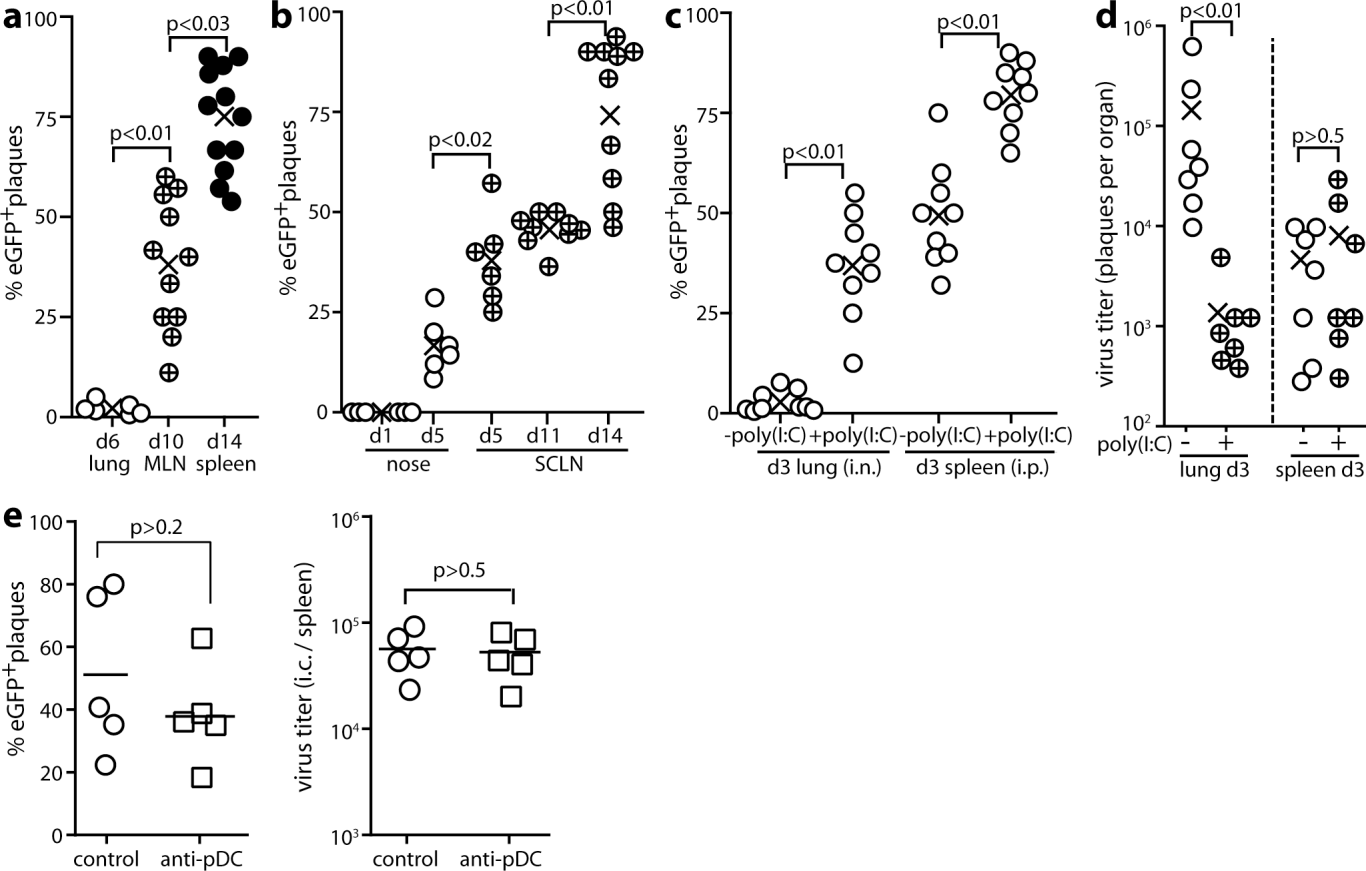
MuHV-4 propagation through IFNαβ-responding cells. **a.** Mx1-cre mice were given MHV-4-RG i.n. (3x10^4^ p.f.u. in 30μl under anesthesia). Viruses were recovered from lungs by plaque assay and from lymphoid tissue by intact cell explant onto BHK-21 cell monolayers. Plaques were then typed as eGFP^+^ (switched) or mCherry^+^ (unswitched). For each mouse (circles), % eGFP^+^ = % switched of total recovered plaques. Crosses show means. MLN = mediastinal lymph nodes. **b.** Mice were given MHV-RG i.n. (3x10^4^ p.f.u. in 5μl without anesthesia) to infect just the nose, then analysed for viral fluorochrome switching as in **a**. SCLN = superficial cervical lymph nodes. Viruses were recovered from noses by plaque assay and from SCLN by intact cell explant (infectious centre assay). **c.** Mice were given i.n. or i.p. MHV-RG, with or without poly(I:C) (50μg i.n. or i.p. 6h before and at the time of infection) to maximally induce IFN-I. Viruses recovered 3d later from lungs by plaque assay (i.n.) or from spleens by infectious centre assay (i.p.) were analysed for fluorochrome expression as in **a**. **d.** Mice were infected i.n. (lung) or i.p. (spleen) and given poly(I:C) or not as in **c**. Virus titers were determined 3d later by plaque assay. Circles show individuals, crosses show means. **e.** Mx1-cre mice were depleted or not of pDCs with mAb 120G8, then infected i.p. with MuHV-RG (10^5^ p.f.u.). 5d later spleens were titered for total recoverable virus by explant of intact splenocytes onto BHK-21 cells. Infectious centres (ICs) were also typed as mCherry^+^ (unswitched) or GFP^+^ (switched). % eGFP^+^ = % of total plaques that were switched. Bars show mean ± SEM, other symbols show individual mice. pDC depletion had no significant effect.

To track how IFN-I responses and viral replication overlap in single cells, we infected Mx1-cre mice, in which cre is transcribed from an IFN-I-inducible Mx1 promoter [35], with MuHV-4 in which cre switches a fitness-neutral (S1 Fig) reporter construct from mCherry (red) to eGFP expression (green) (MHV-RG) [27] (Fig 2). By typing recovered viruses as mCherry^+^ or eGFP^+^ we could determine whether they had passed through a cre^+^ cell; and as Mx1 induction is essentially specific to IFN-I [36–39], this would tell us whether they had passed through an IFN-I-responding cell. MuHV-4 infected Mx1-cre mice equivalently to non-transgenic littermates (S2 Fig). Viruses recovered from the lungs at d6 after i.n. inoculation under anesthesia showed little fluorochrome switching (Fig 2A); those recovered from mediastinal lymph nodes (MLN) at d10 were significantly more switched; and those recovered from spleens at d14 were more switched again. Upper respiratory tract inoculation similarly gave little virus switching in noses at d5; significantly more in superficial cervical LN (SCLN) at d5; and significantly more again in SCLN at d14. Therefore exposure to IFN-I was associated with lymphoid rather than epithelial infection.

As lymphoid infection follows epithelial infection, greater fluorochrome switching in spleens than in lungs could also have reflected time-dependent IFN-I induction. To analyse spleen infection without a preceding lung infection, we gave MHV-RG to Mx1-cre mice i.p., when it reaches splenic MZ macrophages directly—presumably via the thoracic duct and blood [3]. At d3 after i.p. inoculation, splenic virus was significantly more switched than that recovered from lungs at d3 after i.n. inoculation (Fig 2C). This result suggested that viral IFN-I exposure is intrinsically greater in the spleen. Poly(I:C), which is used widely to activate the Mx1-cre transgene, increased virus switching in each site, with again significantly more switching in spleens. It inhibited viral replication only in lungs (Fig 2D). IFN-I responses should drive viral fluorochrome switching but inhibit replication; viral inhibition of IFN-I production or signalling should preserve replication but limit switching; high level switched virus recovery from spleens implied both IFN-I induction and resistance to its effects.

pDCs are important IFN-I producers in some settings [7]. Depleting them with mAb 120G8 (Fig 2E) had no significant effect on the switching or titer of i.p.-inoculated MHV-RG. The same treatment increased significantly the titer of footpad-inoculated MuHV-4 (S3 Fig), so it was functionally effective. Thus, the IFN-I response to spleen infection did not depend strongly on plasmacytoid DCs.

### MuHV-4 resists IFN-I in B cells

**Fig 3:**
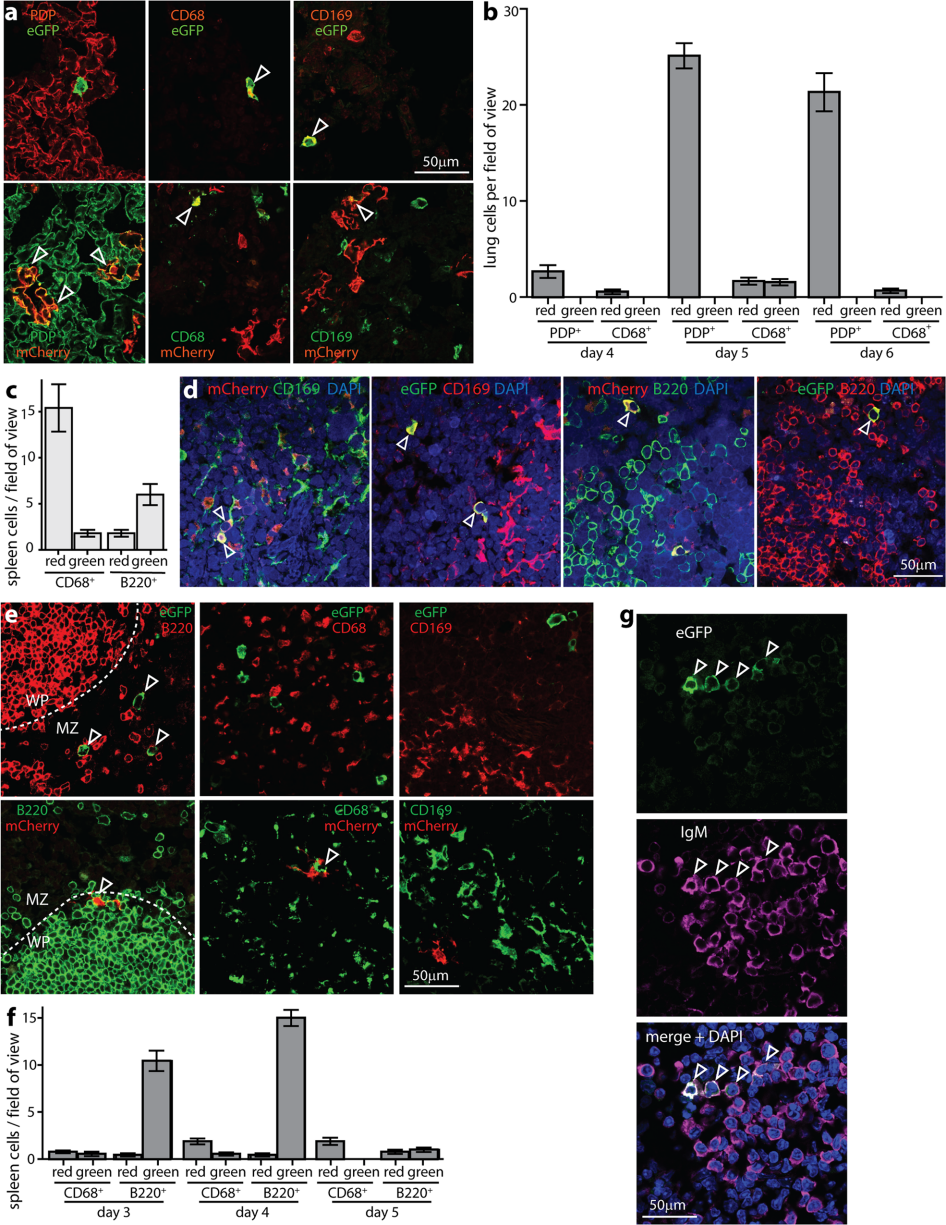
Viral fluorochrome switching in infected lung and spleen cells. **a.** Mx1-cre mice were given i.n. poly(I:C) (50μg) to induce IFN-I 6h before and at the time of i.n. infection with MHV-RG (3x10^4^ p.f.u.). 5d later lung sections were stained for viral eGFP / mCherry, and the cell markers podoplanin (PDP, AEC1s), CD68 (macrophages) and CD169 (AMs). Each image is representative of 5 mice. Arrows show example positive cells. Quantitation is shown in **b**. The cytoplasmic rather than nuclear distribution of eGFP is typical for the relatively mild fixation of periodate-lysine-1% formaldehyde. **b.** Lung sections of mice infected as in **a** (5 per group) were analysed at d4-6, counting eGFP^+^ (green) and mCherry^+^ (red) PDP^+^ and CD68^+^ cells for 3 sections per mouse. Each section corresponds to approximately 10 fields of view as illustrated in **a**. Bars show mean ± SEM. At d5 CD68^+^ cells showed significantly more fluorochrome switching than PDP^+^ cells (p<0.01). **c.** Mx1-cre mice were infected i.p. with MHV-RG (10^5^ pfu), and eGFP^+^ (green) and mCherry^+^ (red) MZ macrophages (CD169^+^) and B cells (B220^+^) counted 4d later. Bars show mean ± SEM counts for 3 sections each of 3 mice per group. B220^+^ cells were significantly more switched than unswitched, whereas CD169^+^ were significantly more unswitched than switched (p<0.02). **d.** Example images of mice infected as in **c**. Arrows show positive cells. **e.** Mx1-cre mice were infected i.p. with MHV-RG (10^5^ pfu), as in **d**, but poly(I:C) (50μg i.p.) was given 6h before and at the time of infection to test the effect of increased IFN-I induction. Spleen sections were analysed at d4 for viral eGFP and mCherry expression in B220^+^ B cells and CD68^+^CD169^+^ MZ macrophages. Arrows show example positive cells. Quantitation is shown in **f**. **f.** Mean ± SEM counts are shown for 3 sections each of 5 mice per group, analysed as in **e** at d3-5 post-infection. Each field of view was 10x the area of the images shown in **e**, and we counted at least 10 fields of view per section (at least 200 viral fluorochrome^+^ cells). B220^+^ cells showed significantly more switched than unswitched fluorochrome expression at d3 and d4 of infection (p<10^−4^). CD68^+^ cells did not (p>0.5). **g.** Mice were infected as in **e** and infected MZ B cells identified by staining for IgM. WP B cells express lower levels of IgM and while this is detectable by flow cytometry, by confocal microscopy as used here, WP B cells are IgD^+^IgM^-^ [3]. >90% of infected MZ B cells were eGFP^+^, confirming the results of **e-f**.

The M3 promoter used to drive MuHV-4 fluorochrome expression is active in the lytic cycle [40]. It is transcribed in infected lungs and acutely infected lymphoid tissue [41, 42], including flow cytometrically sorted lymphoid and myeloid sub-populations [43], and it has revealed AMs and AEC1 colonization in the lungs [29], and MZ macrophage and B cell colonization in the spleen [3]. Therefore it identifies not only any virus reactivating *ex vivo* (Fig 2), but also the cells known to be acutely infected *in vivo* (Fig 3). In infected lungs, >99% of fluorescent cells were unswitched (mCherry^+^eGFP^-^). Even when IFN-I was induced with poly(I:C), essentially all the cells infected at d4 were unswitched. Fig 3A shows examples of staining; Fig 3B shows pooled results. At d5, most fluorescent cells were unswitched AEC1s (mCherry^+^PDP^+^), but of fluorescent AMs (CD68^+^ or CD169^+^) 50% were eGFP^+^. By d6, all fluorescent cells were again mCherry^+^. Therefore MuHV-4 initiated lytic gene expression in IFN-I responding AMs, but the subsequent loss of eGFP^+^ AMs, lack of eGFP spread to AEC1s (Fig 3B) and low switching of recovered virus (Fig 2) argued that these cells poorly supported new virion production. The lack of eGFP^+^ AEC1s after poly(I:C) treatment suggested that these cells made little Mx1 response, although mCherry^+^ cells might still produce eGFP^+^ virions, as switching could occur after viral fluorochrome expression.

Virus tagging is most informative acutely, as the site of tagging is then clearer. Thus to track IFN-I exposure in spleens we gave mice MHV-RG i.p. for direct infection, as in Fig 2C. Fig 3C shows results pooled from multiple sections; Fig 3D shows examples of staining. Fluorescent cells were sparse, but across multiple sections there were significantly more examples of switched than unswitched B cells, and significantly fewer examples of switched than unswitched macrophages. Therefore the switching occurred in B cells, and this virus evidently remained reactivation-competent (Fig 2D).

To test more stringently the capacity of MuHV-4 to resist IFN-I in spleens, we induced it with poly(I:C) (Fig 3E and 3F). Now at d3 50% of fluorescent macrophages were eGFP^+^ (switched), and at d3-4 >90% of fluorescent B cells were eGFP^+^. >80% of these were in the MZ and stained for IgM (Fig 3G). As in lungs, the number of switched macrophages declined after d3, and the number of fluorescent B cells declined after d4, consistent with M3 transcription being silenced as splenic colonization shifts to latency in the white pulp (WP) [3]; but the extensive switching of viruses recovered from spleens at d14 after i.n. infection (Fig 2A and 2B) indicated that IFN-I-exposed genomes remained reactivation-competent.

### MuHV-4 induces IFN-I responses in B cells but not AEC1s

**Fig 4:**
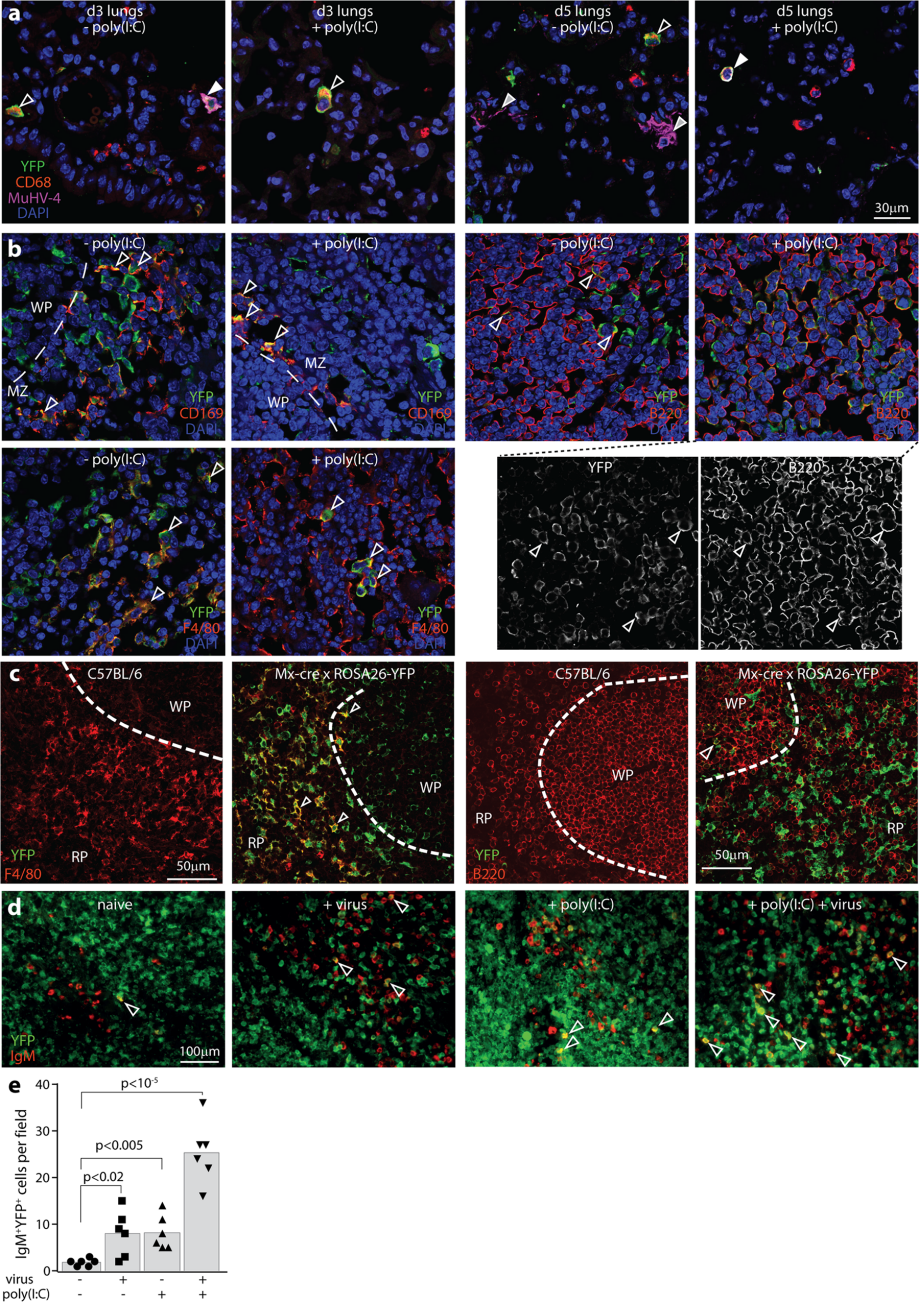
IFNαβ responses measured by Mx1-cre activation of ROSA26-YFP. **a.** Mx1-cre x ROSA26-YFP mice were infected i.n. with MuHV-4 (3x10^4^ p.f.u.). IFN-I was induced or not with poly(I:C) (50μg i.n. and i.p., 6h before and at the time of infection). Lungs were harvested at d3 and d5 and stained for YFP, CD68 and MuHV-4 antigens. Nuclei were stained with DAPI. White arrows show example YFP^+^CD68^+^MuHV-4^+^ AMs; open arrows show YFP^+^CD68^+^ AMs; Grey-filled arrows show YFP^-^MuHV-4^+^ AEC1s. No AEC1s were YFP^+^. All YFP^+^ cells were AMs (n>30). Images are representative of 5 sections from each of 3 mice per group. **b.** Mx1-cre x ROSA26-YFP mice were infected i.p. with MuHV-4 (10^5^ p.f.u.). IFN-I was induced or not with poly(I:C) (50μg i.p., 6h before and at the time of infection). D5 spleen sections were stained for YFP and CD169 (MZ macrophages), F4/80 (RP macrophages) or B220 (B cells). Nuclei were stained with DAPI and the cells visualized by confocal microscopy. Arrows show example YFP^+^ cells expressing the relevant cellular marker. For CD169 staining a dashed line shows the boundary between the B cell-dominated WP and the macrophage-dominated MZ, with CD169^+^ MZ macrophages lying adjacent to WP B cells. For B220 staining after virus + poly(I:C), separate channels are shown to make clear the extensive YFP expression in B cells. Images are representative of 5 sections from each of 3 mice per group. >50% of each macrophage population was YFP^+^, with no difference between poly-IC treated and untreated. In WP follicles, 10–50% of B220^+^ B cells were YFP^+^ after poly(I:C) treatment and 5–20% were YFP^+^ without poly(I:C) treatment. Quantitation for IgM^+^ B cells is shown in **e**. **c.** Naïve C57BL/6 and Mx1-cre x ROSA26-YFP spleen sections were stained for YFP, B220 and F4/80. YFP expression was evident in >80% of F4/80^+^ macrophages and <5% of B220^+^ B cells. Dashed lines show the MZ demarcation between F4/80^+^ RP macrophages and B220^+^ WP B cells. **d.** Mx1-cre x ROSA26-YFP mice were infected as in **b**. Spleen sections were stained for YFP and IgM to identify MZ B cells, and visualized by epifluorescence microscopy. Arrows show example YFP^+^IgM^+^ cells. **e.** Quantitation of YFP^+^IgM^+^ spleen cell numbers across 3 sections from each of 2 mice per group (5 fields of view per section). YFP expression in IgM^+^ B cells was significantly induced by both infection and poly(I:C). Bars show means, other symbols show individual mice.

The Mx1-cre transgene has been used extensively to delete floxed cellular genes via poly(I:C) injection. We reasoned that it could also reveal cellular exposure to IFN-I, via activation of a floxed cellular fluorochrome. To this end we crossed Mx1-cre with ROSA26-YFP mice, in which cre-dependent removal of a floxed translational stop activates YFP expression, and infected them with wild-type MuHV-4 (Fig 4). Infected lungs contained few YFP^+^ cells, even after IFN-I induction with poly(I:C) (Fig 4A). The YFP^+^ cells were all AMs: none showed the characteristic cytoplasmic extensions of AEC1s and YFP failed to co-localize with PDP, despite AEC1s being the main site of lung infection [29]. Thus, viral fluorochrome switching in the lungs was limited both by poor IFN-I induction, and—as IFNβ was readily detected in lung washes after poly(I:C) treatment (Fig 1C)—also by the lack of Mx1 response made by AEC1s. The Mx1 response of lung cells evident in Fig 1D was presumably made by AMs.

Infected spleens contained contrastingly large numbers of YFP^+^ macrophages (F4/80^+^, CD169^+^) and B cells (B220^+^) (Fig 4B). However YFP expression in macrophages was not specific to infection, as it was widespread also in naive (and specific pathogen-free) Mx1-cre x ROSA26-YFP mice (Fig 4C). The original description of Mx1-cre mice showed little spontaneous cre expression [35]. However the marker used—floxed DNA polymerase inactivation—might have been subject to negative selection. Our evidence of constitutive Mx1 transcription in macrophages was consistent with the significant role IFN-I plays in normal myeloid cell differentiation [44]. YFP^+^B220^+^ B cells were contrastingly uncommon in naive Mx1-cre x ROSA26-YFP mice and increased >5-fold by infection. IgM^+^YFP^+^ cell numbers also increased significantly after MuHV-4 infection or poly(I:C) injection (Fig 4D and 4E). Therefore while it was not possible to infer viral exposure to IFN-I in macrophages, cellular fluorochrome switching supported the idea that MuHV-4 is little exposed to IFN-I signalling in AEC1s and abundantly exposed in B cells.

### Viral IFN-I evasion promotes splenic infection

B cell colonization despite viral exposure to IFN-I implied an important role for IFN-I evasion. The MuHV-4 ORF36 inhibits IRF3 signalling, and ORF36^-^ MuHV-4 delivered i.n. shows an IFNAR-dependent infection defect in lungs and spleens [9]. Because spleens are colonized down-stream of lungs, direct and indirect effects on spleen infection are hard to distinguish by i.n. inoculation. Therefore we compared ORF36^-^ MuHV-4 with wild-type also by i.p. inoculation, which reaches the spleen directly [3].

**Fig 5:**
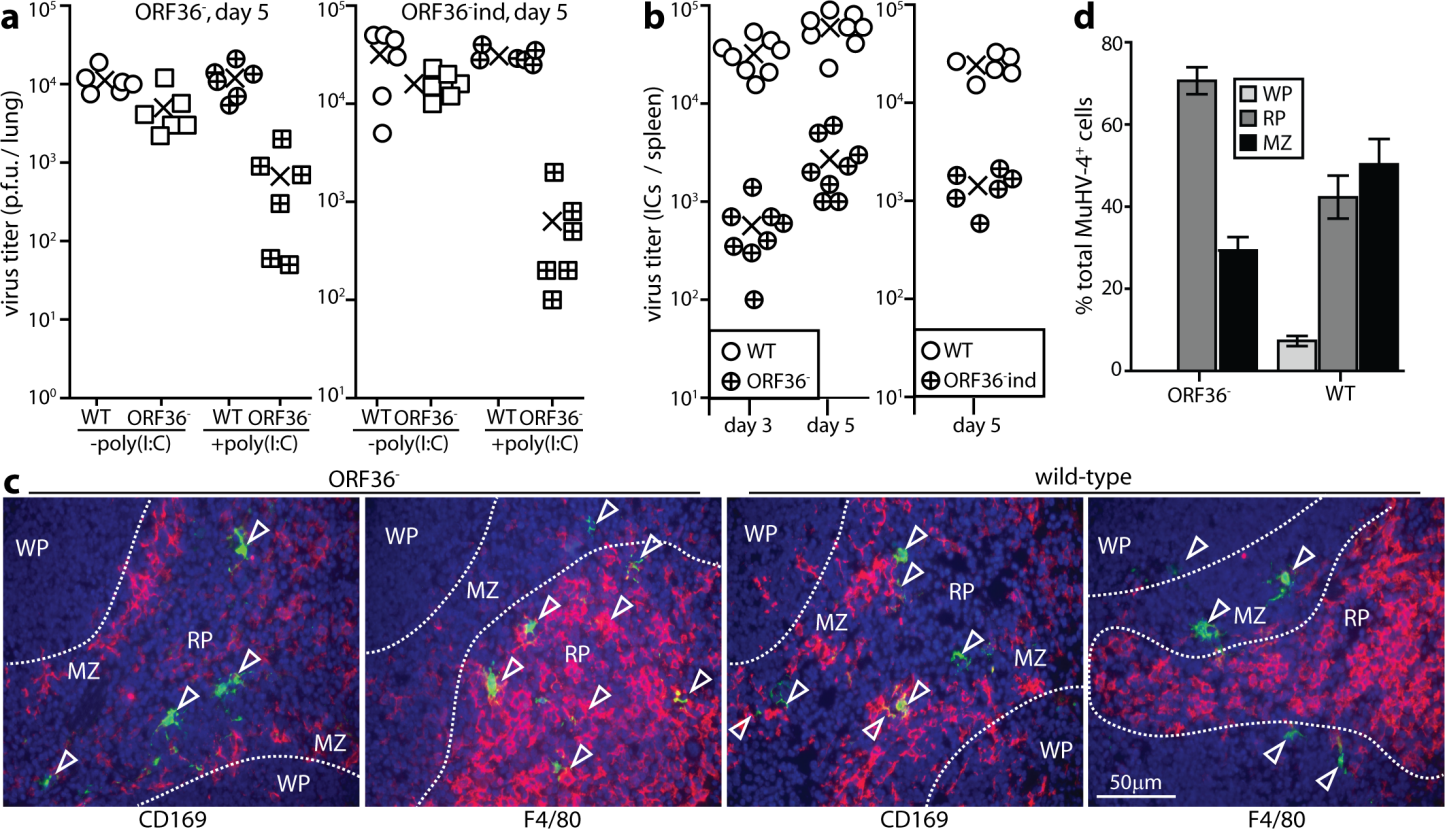
MuHV-4 ORF36 disruption reduces mainly splenic infection. **a.** Mice were infected i.n. with wild-type (WT) or ORF36^-^ MuHV-4 or an independently derived ORF36 mutant (ORF36^-^ind) (3x10^4^ p.f.u.). IFN-I was induced or not with poly(I:C) (50μg i.p. and i.n. 6h before and at the time of infection). Lungs were titered for infectious virus by plaque assay at d5. Crosses show means, other symbols show individual mice. WT titers were not significantly affected by poly(I:C) treatment (p>0.5) whereas ORF36^-^ titers were significantly reduced (p<0.01). **b.** Mice were given WT or ORF36^-^ MuHV-4 i.p. (10^5^ pfu). Explanting intact spleen cells onto BHK-21 cell monolayers yielded significantly fewer ORF36^-^ than WT infectious centres (ICs) at d3 and d5 (p<0.001). Circles show individual mice, crosses show means. **c.** Mice were given ORF36^-^ or WT MuHV-4 i.p. as in **b**. At d4, spleen sections were stained for viral antigens (green) and cellular markers (red). Arrows show example infected RP (F4/80^+^) and MZ macrophages (CD169^+^), with most ORF36^-^ infection being in the RP. **d.** Quantitation of staining as in **c** showed significantly more WT than ORF36^-^ infection in the WP and MZ, and significantly less in the RP (p<0.03). Bars show mean ± SEM (3 sections each of 5 mice per group).

Our ORF36^-^ MuHV-4 showed a relatively minor defect in direct lung infection when given i.n. (Fig 5A), and a marked defect in direct spleen infection when given i.p. (Fig 5B). IFN-I induction with poly(I:C) increased the lung infection defect (Fig 5A). Thus in lungs, limited IFN-I induction normally allows ORF36^-^ MuHV-4 to achieve near wild-type titers; but in spleens, where IFN-I induction was greater, ORF36 was an important outcome determinant. Poor splenic infection by ORF36^-^ MuHV-4 was associated with a shift in viral antigen from MZ (CD169^+^) to red pulp (RP, F4/80^+^) macrophages (Fig 5C and 5D). Impaired virus transfer to the WP provided an explanation for the reduction in titer, as lymphoproliferation in the WP normally amplifies the viral load. Virus deposition in the RP was consistent with virions being carried by blood flow from the MZ when transfer to the WP was impaired.

### IFN-I blockade increases MuHV-4 replication in macrophages

**Fig 6:**
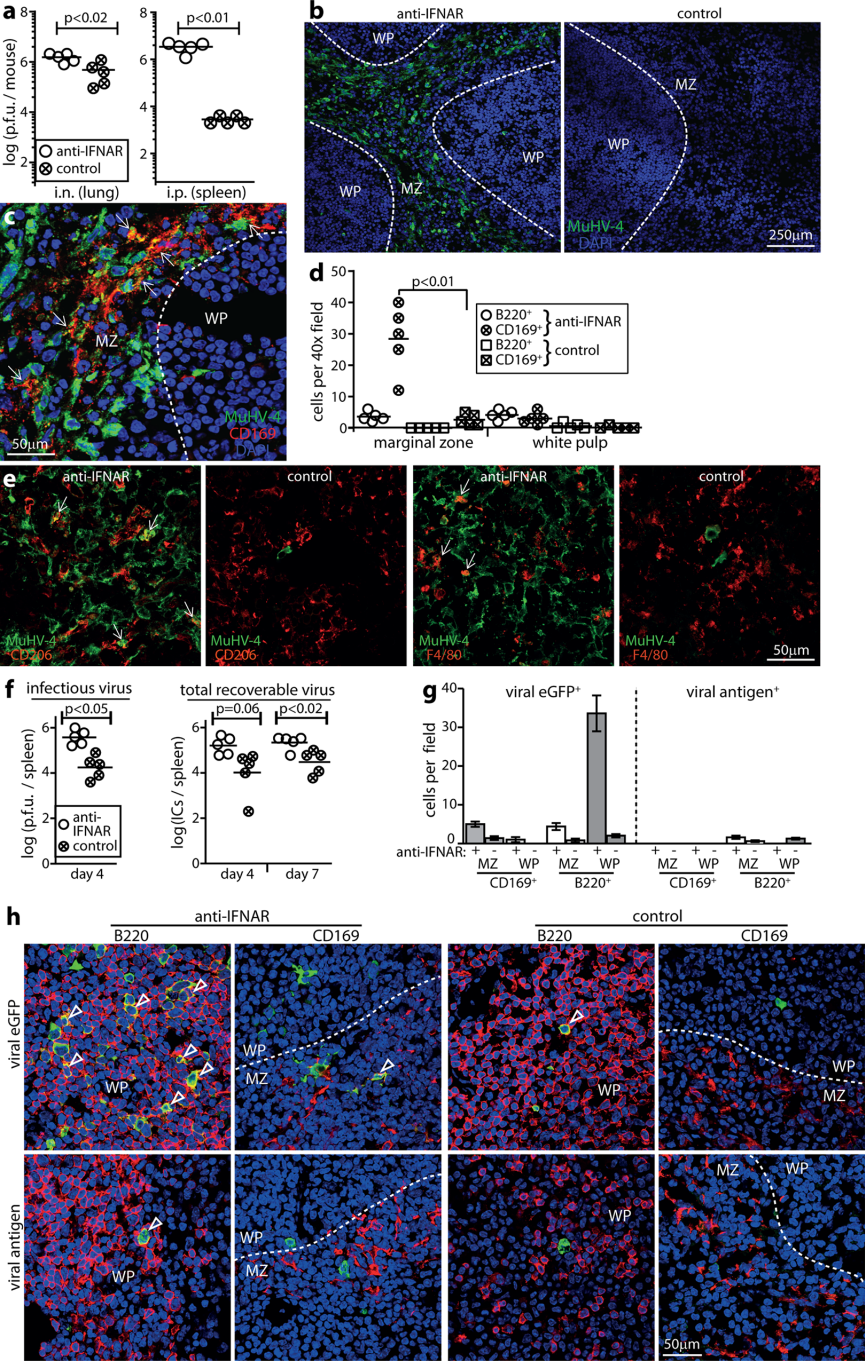
Effect of IFNAR blockade on MuHV-4 infection of MZ macrophages and WP B cells. **a.** C57BL/6 mice were given an anti-IFNAR blocking antibody i.p. or not (control) then infected i.n. or i.p. with wild-type MuHV-4. Virus in lungs (i.n.) or spleens (i.p.) was titered by plaque assay after 4d. Circles show individuals, bars show means. The IFNAR blockade significantly increased virus titers in both sites, but the difference was significantly greater in spleens (p<0.01). **b.** Spleens of mice infected i.p. as in **a** were stained for MuHV-4 antigens. Nuclei were stained with DAPI. Dashed lines show approximate MZ / WP boundaries, based on CD169 staining of adjacent sections. IFNAR blockade increased MZ infection >100 fold and WP infection <5-fold. In the MZ >90% of viral antigen^+^ cells had typical myeloid cell rather than B cell morphology. **c.** Co-staining of a representative anti-IFNAR-treated, i.p.-infected mouse after 4d shows extensive viral antigen expression in CD169^+^ splenic MZ macrophages (arrows). Quantitation is shown in **d**. **d.** Quantitation of MZ and WP viral antigen staining for spleen sections from 5 mice per group, treated or not with anti-IFNAR antibody and infected i.p. as in **a**. Bars show group means, other symbols show mean counts for 3 sections per mouse. IFNAR blockade significantly increased viral antigen expression in all populations (p<0.05), most markedly in CD169^+^ MZ macrophages (p<0.01). **e.** Spleen sections of mice infected as in **b**, showing additional co-localization of viral antigen with CD206^+^ and F4/80^+^ macrophages after IFNAR blockade (arrows). Again IFNAR blockade significantly increased MuHV-4 antigen expression in these populations relative to no antibody controls (p<0.01). **f.** Mice were given anti-IFNAR antibody or not (control) and 2d later infected i.p. with MHV-GFP. Spleens were titered for infectious virus by plaque assay after 4d and for total reactivatable virus by infectious centre (IC) assay after 4 and 7d. Circles show individuals, horizontal bars show means. **g.** Spleens of mice infected as in **f** were analysed at d7 for viral eGFP and antigen expression in CD169^+^ macrophages and B220^+^ B cells. Bars show mean ± SEM of 5 mice. IFNAR blockade significantly increased viral eGFP expression in all populations (p<0.05), most markedly in WP B cells (p<10^−4^). **h.** Example images of mice infected as in **f**, stained for viral eGFP (green) and cell markers (red). Nuclei were stained with DAPI (blue). The dashed lines show approximate WP / MZ boundaries, based on CD169 staining. Arrows show example eGFP^+^ cells. Quantitation is shown in **g**.

Although MuHV-4 colonizes IFN-I-responding mice, i.n. and i.p. inoculations are more pathogenic in IFNAR^-/-^ mutants [16, 45], so IFN-I must normally exert some restriction on virus replication. Which cell types support the additional infection when IFN-I is lacking has been unclear. Blocking IFN-I signalling with an IFNAR-specific antibody increased acute lung infection <5-fold (Fig 6A), consistent with little IFN-I induction in this site (Fig 1); by contrast spleen increased infection >50-fold. Immunostaining revealed extensive viral lytic spread through the splenic MZ and RP, with increased viral antigen expression in cells morphologically typical of macrophages (Fig 6B). Splenic macrophages are diverse and lack a single unifying marker. Viral antigens were evident in CD169^+^ (MZ macrophage) (Fig 6C and 6D), CD206^+^ (tissue resident, non-MZ macrophage) and F4/80^+^ (RP macrophage) populations (Fig 6E), so IFNAR blockade made many macrophage subtypes more permissive for lytic infection. Viral staining in the WP remained low (Fig 6D). Thus in the absence of IFN-I, MZ to WP transfer became rate-limiting for virus spread, and most infection was diverted to the RP, again consistent with untransferred MZ virus following splenic blood flow.

We tracked latent infection in spleens by viral eGFP expression from a constitutive promoter (Fig 6F). Again blocking IFNAR increased infectious virus titers at d4. Total recoverable virus (infectious centre assay) increased similarly to infectious virus, consistent with most early spleen infection being lytic in MZ macrophages [46]. By d7 after i.p. challenge, when spleen infection is largely latent in B cells [46], virus titers remained elevated above controls in IFNAR-blocked mice; however the elevation was no greater than at d4 (Fig 6F), implying that IFN-I limited mainly MZ macrophage infection. Staining spleen sections at d7 (Fig 6G and 6H) showed more WP infection (eGFP^+^) in IFNAR-blocked mice than in controls, so more lytic infection in MZ macrophages eventually fed through to more latent infection in WP B cells. However IFN-I blockade did not increase viral antigen^+^/ viral eGFP^+^ cell ratios in the WP at d7—in fact they were reduced—so the proportion of lytic infection in WP B cells did not increase. We conclude that splenic macrophage infection was strongly restricted by IFN-I, but that acute viral reactivation in WP B cells was largely IFN-I-independent.

IFN-I principally targets viral lytic infection. Therefore it possibly had least effect on B cell infection because this is mostly latent. MuHV-4 that cannot shut down lytic infection due to an additional promoter element inserted upstream of its ORF50 lytic switch gene (M50) replicates normally in mice for 3d, but is then progressively attenuated [47]. This virus cannot drive B cell proliferation because it is constitutively lytic, but it is attenuated also in the lungs, where AEC1s and alveolar macrophages are infected. That IFNAR deficiency does not significantly increase MuHV-4 lung infection for at least 4d [16] suggested that M50 virus attenuation might be due to impaired IFN-I evasion. To test this hypothesis we gave mice IFNAR blocking antibody i.p. then wildtype or M50 MuHV-4 i.n. (10^5^ p.f.u.). Lung virus titers after 7d showed restoration of the M50 replication defect by IFN-I blockade (S4 Fig). Thus, a capacity to establish latency was important for IFN-I evasion.

### Murine cytomegalovirus also passes through IFN-I-responding cells

**Fig 7:**
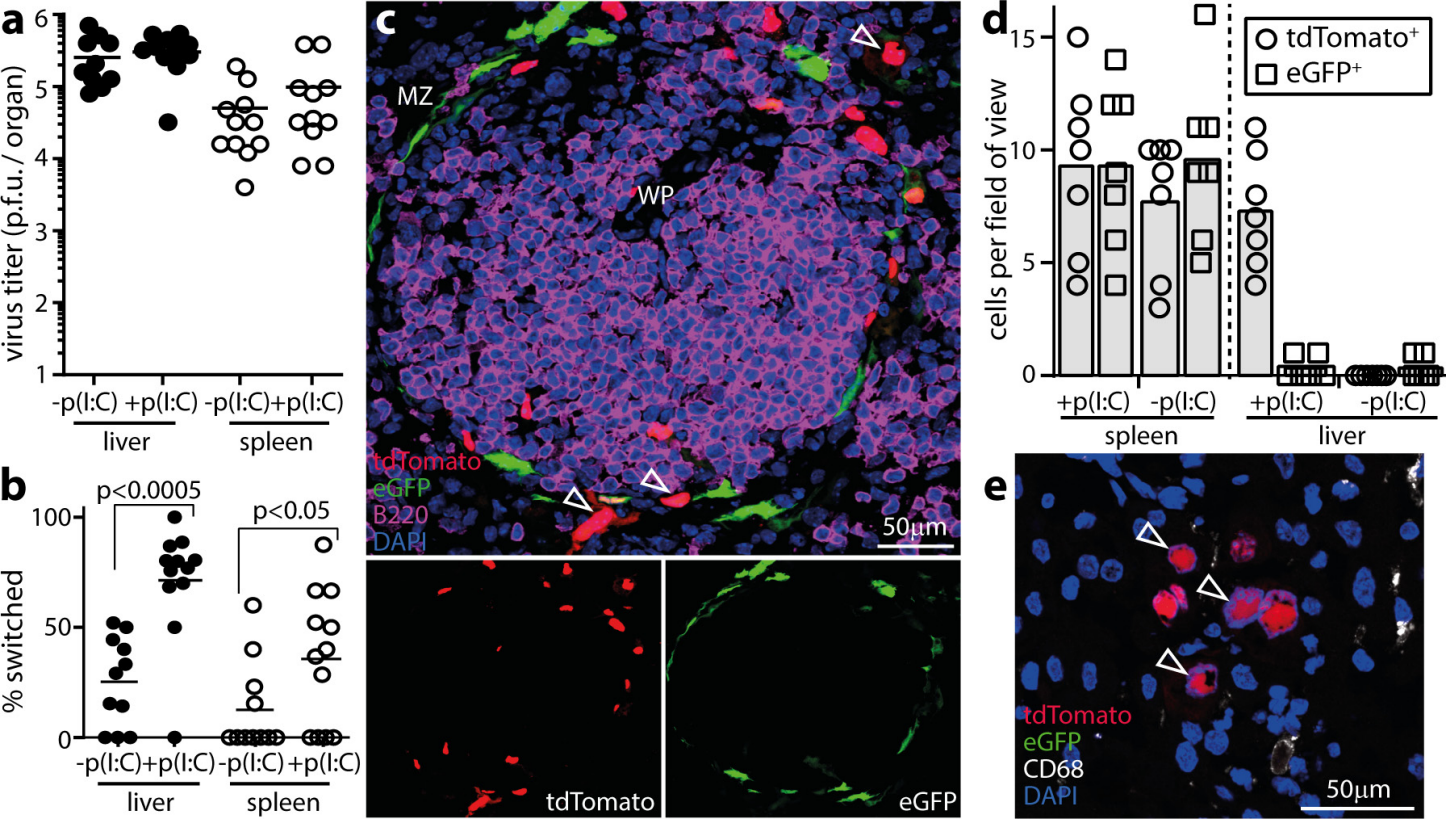
Mx1-cre-dependent fluorochrome switching of MCMV. **a.** Mx1-cre mice were infected i.p. with MCMV-GR, which switches fluorochrome expression when exposed to cre recombinase. IFN-I was induced or not by i.p. poly(I:C) inoculation (pIC, 50μg / mouse) 6h before and at the time of infection. 5d later virus in livers and spleens was titered by plaque assay. Circles show individuals, bars show means. Poly(I:C) had no significant effect on titers (p>0.5). **b.** The samples from **a** were assayed for viral fluorochrome switching by identifying plaques as eGFP^+^ or tdTomato^+^. **c.** Example image from a MCMV-GR-infected and poly(I:C)-treated spleen shows eGFP^+^ and tdTomato^+^ cells around a WP follicle. Quantitation is shown in **d**. **d.** Quantitation of eGFP^+^ and tdTomato^+^ cells on liver and spleen sections, pooled from 3 mice per group infected as in **a**. Bars show means, other symbols show individual sections. Poly(I:C) significantly increased infected cell fluorochrome switching in livers (p<0.01) but not in spleens (p>0.5). **e.** Example image of a MCMV-GR-infected, poly(I:C)-induced, liver showing only tdTomato^+^ cells that do not co-localize with CD68. Two other livers gave equivalent results.

MuHV-4 passage through IFN-I responding cells was surprising: we expected that either viral evasion would prevent an IFN-I response in infected cells, or IFN-I would prevent viral replication. To test whether it was a unique to MuHV-4, we infected Mx1-cre mice with murine cytomegalovirus that cre switches from eGFP to tdTomato expression (MCMV-GR). We induced IFN-I or not with poly(I:C), gave MCMV-GR i.p., and recovered infectious virus from livers and spleens by plaque assay at d3 (Fig 7A). As with MHV-RG, IFN-I induction with poly(I:C) increased MCMV-GR switching (Fig 7B) without reducing titers. Thus MCMV, which like MuHV-4 inhibits IFN-I induction and signalling [48], could also pass through IFN-I-responding cells. Spleen sections (Fig 7C and 7D) revealed eGFP^-^tdTomato^+^ cells around lymphoid follicles, consistent with MCMV infecting IFN-I-responding MZ macrophages [49]. Liver sections of (Fig 7D and 7E) showed CD68^-^tdTomato^+^ cells with the morphology of hepatocytes. Infected cells were more switched than recovered virions, consistent with IFN-I exerting some restriction on virion production [50], but productive infection was clearly possible in IFN-I-responding cells.

## Discussion

MuHV-4 provides an experimental window onto the γ-herpesviruses, whose colonization of lymphoid tissue directly confronts host immune defences. IFN-I attenuated macrophage infection, whereas B cell infection was protected by viral evasion. This cell type-dependent outcome explained how IFN-I and its evasion both control infection, with each dominating in a different setting (S5 Fig). Together they played a significant role in shifting the focus of viral tropism from macrophages acutely to B cells chronically.

In the lungs, AMs provide MuHV-4 with a gateway to AEC1s, which then support lytic replication [29]. AEC1s made no detectable Mx1 response to infection or to poly(I:C). Thus, although IFN-I responding AMs poorly supported virus spread, inducing or blocking IFN-I or disrupting its evasion all had modest effects because AEC1s still allowed virus replication. Splenic MZ macrophages provide a gateway to MZ B cells. Again IFN-I restricted virus spread from macrophages. Unlike AEC1s, B cells made Mx1 responses, but their IFN-I responses were bypassed by viral evasion.

An important role for the ORF36 IFN-I evasion gene in splenic infection was surprising, as its inhibition of IRF3 should limit IFN-I induction, and the abundant exposure of wild-type splenic virus to IFN-I implied that MuHV-4 evades its effector functions rather than relying solely on limiting induction. A possible explanation is that ORF36, as a lytic gene, operates more in macrophages than in B cells, as macrophages support lytic infection [43] and are important IFN-I producers, whereas B cells support a more tightly latent infection and function more prominently as IFN-I responders. IFN-I production by macrophages depends on positive feedback through IFNAR [51]. Thus ORF36, by limiting IFN-I production, should limit IFN-I signalling by infected macrophages both to themselves and to B cells. This would promote macrophage to B cell virus transfer: first, by promoting new virion production in macrophages; and second, by reducing the effector evasion required for those virions to productively enter B cells. The importance for splenic infection of IFNAR degradation by ORF54 [12] suggests that it may play a similar role. Plasmacytoid DC produce IFN-I independently of feedback through IFNAR [7], so their production would not be affected by ORF36 or ORF54. They (B220^+^ DC) are not an acute infection target in spleens [3]. However they contributed relatively little to virus switching or control, so other evasion mechanisms may limit their scope.

The recovery of fluorochrome-switched virions implied that viral IFN-I evasion also operates down-stream of IFN-I induction or signalling, that is downstream of ORF36. Switching was evident early after i.p. virus inoculation, when splenic infection is mostly lytic [46]. Established lytic infection would inhibit IFN-I signalling, so we envisage that most switching occurred when virions entered cells already making IFN-I responses. In this context the disassembly of IFN-I induced ND10 domains [52] may be an important evasion mechanism. This is a function of the MuHV-4 ORF75c tegument component [53, 54] and is conserved in the homologous Kaposi's Sarcoma-associated Herpesvirus ORF75 [55] and Epstein-Barr virus BNRF1 [56]. Tagged viral genomes could then remain latent until IFN-I responses have subsided [14]. Therefore, *pace* cell differences in IFN-I response, macrophage infection may be more susceptible than B cell infection to inhibition by IFN-I because it is more lytic [43, 46].

Together the IFN-I response and its evasion promoted B cell over macrophage infection, making IFN-I an important determinant of *in vivo* viral tropism. A corollary is that IFN-I-based therapies are likely to have only a small window of efficacy—mainly reducing acute gamma-herpesvirus replication in myeloid cells. Downstream of this, virions entering IFN-I-exposed B cells would still establish a viable infection. IFN-I deficiency increases *ex vivo* MuHV-4 reactivation rates [14]. However the explant reactivation assay is complicated, relying on plaque formation in complex *in vitro* cultures that contain both virus-infected and immune cells. Reactivation rates are low, particularly from B cells [43], and may be affected by cell viability, *in vitro* antibody production and cytokines. The failure of IFNAR blockade to increase the viral antigen / eGFP staining ratio of d7 splenic WP B cells argued that here IFN-I is not a major regulator of *in vivo* lytic reactivation. The capacity of MCMV also to re-emerge acutely after Mx1-dependent tagging suggested that many herpesviruses can enter IFN-I responding cells and rapidly re-emerge. How far IFN-I can restrict MCMV was not explored, but clearly there are limits on what it alone is likely to achieve.

## Materials and Methods

### Mice

BALB/c, C57BL/6J and Mx1-cre mice [35] were maintained at University of Queensland animal units. Mx1-cre x ROSA26-YFP mice were bred at the Walter and Eliza Hall Institute. Mice were infected with MuHV-4 or MCMV when 6–8 weeks old, either i.n. (3x10^4^ p.f.u.) under isofluorane anesthesia or i.p. (10^5^ p.f.u.). Luciferase^+^ MuHV-4 infection (MHV-LUC) [42] was imaged by i.p. injection of D-luciferin (2mg, Pure Science) and charge-coupled device camera scanning (IVIS spectrum, Xenogen). IFNαβR signalling was blocked by i.p. injection of mAb MAR-5A3 (100μg/mouse every 2d); pDCs were depleted by i.p. injection of mAb 120G8 (200μg/mouse every 2d) (Bio X Cell). Poly-inosinic/cytidylic acid (poly(I:C), 50μg) was given i.p. (for i.p. infection) or i.n. plus i.p. (for i.n. infection) 6h before and at the time of virus inoculation. Statistical comparison was by Student's 2 tailed unpaired t test unless otherwise stated.

### Ethics

All animal experiments were approved by the University of Queensland and Walter and Eliza Hall Animal Ethics Committees in accordance with Australian National Health and Medical Research Council (NHMRC) guidelines. Project 301/13.

### Cells and viruses

Bovine Hamster Kidney (BHK-21) fibroblasts (American Type Culture Collection CCL-10), RAW-264 monocytes (American Type Culture Collection TIB-71), NIH-3T3 cells (American Type Culture Collection CRL-1658), NIH-3T3-cre cells [57] and fibroblasts (from d13-14 mouse embryos) were grown in Dulbecco’s Modified Eagle’s Medium with 2 mM glutamine, 100 IU/ml penicillin, 100 μg/ml streptomycin, and 10% fetal calf serum (complete medium). All MuHV-4 variants were derived from a BAC-cloned viral genome [58]. ORF36-deficient MuHV-4 was made by shuttle mutagenesis, inserting into an *Xcm*I site (nucleotide 53027 of Genbank sequence NC001826) of the ORF36 coding sequence (52848–54161) an oligonucleotide with multiple stop codons and an *Eco*RI restriction site. Correct mutagenesis was identified by *Eco*RI digestion of BAC DNA, and confirmed by sequencing of viral DNA across the insertion site. Infectious virus was recovered by BAC DNA transfection into BHK-21 cells, and the loxP-flanked BAC cassette removed by virus passage through NIH-3T3-cre cells. Luciferase^+^ [42], floxed reporter (MHV-RG) [27], and eGFP^+^ MuHV-4 [59] are described. MuHV-4 was grown and titered on BHK-21 cells. Floxed reporter MCMV (MCMV-GR) [60] was grown on NIH-3T3 cells. Virions were harvested from infected cell culture by ultracentrifugation (30,000 x *g*, 120min) and cell debris was removed by low speed centrifugation (500 x *g*, 10min).

### Virus assays

To titer infectious virus, culture-grown stocks or freeze-thawed organ homogenates were plated on BHK-21 (MuHV-4) or embryonic fibroblast (MCMV) monolayers [61]. To titer total reactivatable MuHV-4, organs were disrupted into single cell suspensions then plated on BHK-21 cells. The cells were cultured in complete medium for 3h, overlaid with complete medium plus 0.3% carboxymethylcellulose, cultured for 4d, then fixed with 1% formaldehyde and stained with 0.1% toluidine blue. To measure viral fluorochrome switching, plaque assays were performed at limiting dilution, with 16 replicate wells per dilution. After 4d wells were scored for green (eGFP) and red (mCherry or tdTomato) fluorescence to derive virus titers for each colour., with % switching = 100 x switched plaque titer / (switched plaque titer + unswitched plaque titer).

### IFNαβ assays

Murine IFNβ was assayed by ELISA (PBL Verikine). Mx1 mRNA was quantitated in lung tissue (Aurum RNA isolation kit, Bio-Rad) by quantitative PCR (iTaq universal SYBR green kit, Bio-Rad) with Mx1-specific primers (qMmuCID0023356, Bio-Rad), and normalized by parallel amplification of Nidogen-1 (Rotor-Gene, Qiagen).

### Immunostaining

Organs were fixed in 1% formaldehyde / 10 mM sodium periodate / 75 mM L-lysine (18h, 4°C), equilibrated in 30% sucrose (24h, 4°C), then frozen in OCT. Sections (6μm) were air-dried (1h, 23°C), washed 3x in PBS, blocked with 0.3% Triton X-100 / 5% normal donkey serum (1h, 23°C), then incubated (18h, 4°C) with combinations of antibodies to eGFP (rabbit, chicken or goat pAb), CD68 (rat mAb, FA-11) (AbCam), B220 (rat mAb RA3-6B2), F4/80 (rat mAb CI:A3–1) (Santa Cruz Biotechnology), mCherry (rabbit pAb, Badrilla), CD206 (rat mAb MR5D3), CD169 (rat mAb 3D6.112) (Serotec), podoplanin (goat pAb, R&D Systems), and MuHV-4 (polyclonal rabbit sera raised by 2 subcutaneous virus inoculations). Sections were washed 3× in PBS, incubated (1h, 23°C) with combinations of Alexa568-donkey anti-rat IgG pAb, Alexa488 or Alexa647-donkey anti rabbit IgG pAb, Alexa647-donkey anti-mouse IgM pAb, Alexa488-donkey anti-chicken IgG pAb (Abcam), and Alexa488-donkey anti-goat pAb (Life Technologies), then washed 3× in PBS, stained with DAPI and mounted in Prolong Gold (Life Technologies). TdTomato fluoresence was visualized directly. Images were captured with a Zeiss LCM510 confocal microscope or a Nikon epifluorescence microscope and analyzed with Zen imaging software or ImageJ.

## Supporting Information

S1 Fig Normal replication of fluorochrome switching MuHV-4. C57BL/6 mice were infected in lungs (10^4^ p.f.u. i.n. in 30μl under anesthesia) with wild-type MuHV-4 (MHV-WT), colour switching MuHV-4 (MHV-RG), or the same virus pre-switched *in vitro* by passage through cre^+^ NIH-3T3 cells (MHV-G). Lungs were titered for infectious virus after 6 days by plaque assay. Spleens were titered for total recoverable virus after 13 days by infectious center assay. Crosses show means, other symbols show individual mice. No significant difference was seen between MHV-WT, MHV-RG and MHV-G.
(PDF)

S2 Fig Normal MuHv-4 replication in Mx1-cre mice. Mx1-cre mice were bred as heterozygote x non-transgenic. Mx1-cre^+^ heterozygote progeny (Mx1-cre) and non-transgenic littermates (C57BL/6) were infected i.n. when 6–8 weeks old with wild-type MuHV-4 (10^4^ p.f.u. i.n. in 30μl under isoflurane anesthesia). Infectious virus in lungs was titered after 6 days by plaque assay, and lytic plus latent virus in spleens was titered after 13 days by infectious center assay. Crosses show means; other symbols show individual mice. No significant difference was observed between Mx1-cre^+^ and C57BL/6 mice.
(PDF)

S3 Fig Functional effect of plasmacytoid dendritic cell (pDC) depletion. Mice were gives anti-pDC mAb (3x400μg, mAb BX444, anti-CD317/BST2/PDCA-1, Bio X Cell) or not i.p., then MuHV-4 into footpads (10^5^ p.f.u.). 3 days later footpads, popliteal lymph nodes (PLN) and spleens were titered for virus by infectious center assay. Bars show mean ± SEM for 3–6 mice. Virus titers were significantly reduced in footpads by Student’s unpaired 2 tailed t test, but not in PLN or spleens (ns = not significant, p>0.05).
(PDF)

S4 Fig IFNAR-dependent attenuation of MuHV-4 with increased lytic reactivation. Mice were given anti-IFNAR blocking mAb (100μg i.p. every other day, αIFN) or not (nil) then wild-type (WT) or M50 MuHV-4 i.n. (10^5^ p.f.u.). M50 MuHV-4 has the proximal 416bp of the Murine cytomegalovirus IE1 promoter inserted in its ORF50 exon1 5’ untranslated region. ORF50 encodes the MuHV-4 lytic switch protein. M50 MuHV-4 shows increased ORF50 transcription and an incapacity to remain latent (May JS, Coleman HM, Smillie B, Efstathiou S, Stevenson PG (2004) Forced lytic replication impairs host colonization by a latency-deficient mutant of murine gammaherpesvirus-68. J Gen Virol 85: 137–146). At 7 days after infection, lungs were titered for infectious virus by plaque assay. Crosses show means, other symbols show individual mice. Without aIFN mAb M50 titers were significantly less than wild-type (p<0.001 by Student’s unpaired 2-tailed test); with IFNAR blockade M50 and WT titers were not significantly different.
(PDF)

S5 Fig Summary of how IFN-I and MuHV-4 replication interact in different infected cell types. Type 1 alveolar epithelial cells made no detectable Mx1 response to MuHV-4 infection or to p(I:C), and IFN-I induction had little effect on viral replication in the lungs, where these cells are abundantly infected. Thus, their interaction was dominated by poor responsiveness to IFN-I. Macrophages contrastingly showed viral fluorochrome switching but propagated switched virions poorly, and IFN-I blockade increased massively the extent of their infection. Thus, in macrophages IFN-I was protective. B cells were different again. They showed abundant viral fluorochrome switching and switched virion production. IFN-I blockade had little effect on infection, but viral evasion gene disruption caused marked attenuation, indicating that B cell infection is normally dominated by IFN-I evasion. This implies that virions can enter IFN-I-responding B cells and establish a latent infection that is stably maintained and can reactivate, presumably when IFN-I signalling has diminished.
(PDF)

## References

[1] Sunil-Chandra NP, Efstathiou S, Nash AA (1992) Murine gammaherpesvirus 68 establishes a latent infection in mouse B lymphocytes in vivo. J Gen Virol 73: 3275–3279. 1469366 10.1099/0022-1317-73-12-3275

[2] Gaspar M, May JS, Sukla S, Frederico B, Gill MB, et al (2011) Murid herpesvirus-4 exploits dendritic cells to infect B cells. PLoS Pathog 7: e1002346 10.1371/journal.ppat.1002346 22102809 PMC3213091

[3] Frederico B, Chao B, May JS, Belz GT, Stevenson PG (2014) A murid gamma-herpesviruses exploits normal splenic immune communication routes for systemic spread. Cell Host Microbe 15: 457–470. 10.1016/j.chom.2014.03.010 24721574

[4] Frederico B, Milho R, May JS, Gillet L, Stevenson PG (2012) Myeloid infection links epithelial and B cell tropisms of Murid Herpesvirus-4. PLoS Pathog 8: e1002935 10.1371/journal.ppat.1002935 23028329 PMC3447751

[5] Schultz U, Kaspers B, Staeheli P (2004) The interferon system of non-mammalian vertebrates. Dev Comp Immunol 28: 499–508. 15062646 10.1016/j.dci.2003.09.009

[6] Kawai T, Akira S (2006) Innate immune recognition of viral infection. Nat Immunol 7: 131–137. 16424890 10.1038/ni1303

[7] Colonna M, Trinchieri G, Liu YJ (2004) Plasmacytoid dendritic cells in immunity. Nat Immunol 5: 1219–1226. 15549123 10.1038/ni1141

[8] Randall RE, Goodbourn S (2008) Interferons and viruses: an interplay between induction, signalling, antiviral responses and virus countermeasures. J Gen Virol 89: 1–47. 18089727 10.1099/vir.0.83391-0

[9] Hwang S, Kim KS, Flano E, Wu TT, Tong LM, et al (2009) Conserved herpesviral kinase promotes viral persistence by inhibiting the IRF-3-mediated type I interferon response. Cell Host Microbe 5: 166–178. 10.1016/j.chom.2008.12.013 19218087 PMC2749518

[10] Kang HR, Cheong WC, Park JE, Ryu S, Cho HJ, et al (2014) Murine gammaherpesvirus 68 encoding open reading frame 11 targets TANK binding kinase 1 to negatively regulate the host type I interferon response. J Virol 88: 6832–6846. 10.1128/JVI.03460-13 24696485 PMC4054366

[11] Liang X, Shin YC, Means RE, Jung JU (2004) Inhibition of interferon-mediated antiviral activity by murine gammaherpesvirus 68 latency-associated M2 protein. J Virol 78: 12416–12427. 15507628 10.1128/JVI.78.22.12416-12427.2004 PMC525078

[12] Leang RS, Wu TT, Hwang S, Liang LT, Tong L, et al (2011). The anti interferon activity of conserved viral dUTPase ORF54 is essential for an effective MHV-68 infection. PLoS Pathog 7, e1002292 10.1371/journal.ppat.1002292 21998588 PMC3188543

[13] Sheridan V, Polychronopoulos L, Dutia BM, Ebrahimi B (2014) A shutoff and exonuclease mutant of murine gammaherpesvirus-68 yields infectious virus and causes RNA loss in type I interferon receptor knockout cells. J Gen Virol 95: 1135–1143. 10.1099/vir.0.059329-0 24552788

[14] Mandal P, Krueger BE, Oldenburg D, Andry KA, Beard RS, et al (2011) A gammaherpesvirus cooperates with interferon-alpha/beta-induced IRF2 to halt viral replication, control reactivation, and minimize host lethality. PLoS Pathog 7: e1002371 10.1371/journal.ppat.1002371 22114555 PMC3219715

[15] Goodwin MM, Canny S, Steed A, Virgin HW (2010) Murine gammaherpesvirus 68 has evolved gamma interferon and stat1-repressible promoters for the lytic switch gene 50. J Virol 84: 3711–3717. 10.1128/JVI.02099-09 20071569 PMC2838114

[16] Dutia BM, Allen DJ, Dyson H, Nash AA (1999). Type I interferons and IRF-1 play a critical role in the control of a gammaherpesvirus infection. Virology 261, 173–179. 10497103 10.1006/viro.1999.9834

[17] Barton ES, Lutzke ML, Rochford R, Virgin HW (2005). Alpha/beta interferons regulate murine gammaherpesvirus latent gene expression and reactivation from latency. J. Virol. 79, 14149–14160. 16254350 10.1128/JVI.79.22.14149-14160.2005 PMC1280204

[18] Cardin RD, Brooks JW, Sarawar SR, Doherty PC (1996) Progressive loss of CD8+ T cell-mediated control of a gamma-herpesvirus in the absence of CD4+ T cells. J Exp Med 184: 863–871. 9064346 10.1084/jem.184.3.863 PMC2192775

[19] Jacoby MA, Virgin HW, Speck SH (2002) Disruption of the M2 gene of murine gammaherpesvirus 68 alters splenic latency following intranasal, but not intraperitoneal, inoculation. J Virol 76: 1790–1801. 11799175 10.1128/JVI.76.4.1790-1801.2002 PMC135904

[20] Macrae AI, Usherwood EJ, Husain SM, Flaño E, Kim IJ, et al (2003). Murid herpesvirus 4 strain 68 M2 protein is a B-cell-associated antigen important for latency but not lymphocytosis. J Virol 77: 9700–9709. 12915582 10.1128/JVI.77.17.9700-9709.2003 PMC187398

[21] Simas JP, Marques S, Bridgeman A, Efstathiou S, Adler H (2004) The M2 gene product of murine gammaherpesvirus 68 is required for efficient colonization of splenic follicles but is not necessary for expansion of latently infected germinal centre B cells. J Gen Virol 85: 2789–2797. 15448339 10.1099/vir.0.80138-0

[22] Marques S, Alenquer M, Stevenson PG, Simas JP (2008) A single CD8+ T cell epitope sets the long-term latent load of a murid herpesvirus. PLoS Pathog 4: e1000177 10.1371/journal.ppat.1000177 18846211 PMC2556087

[23] Stevenson PG, Simas JP, Efstathiou S (2009) Immune control of mammalian gamma-herpesviruses: lessons from murid herpesvirus-4. J Gen Virol 90: 2317–2330. 10.1099/vir.0.013300-0 19605591

[24] Sancho-Shimizu V, Perez de Diego R, Jouanguy E, Zhang SY, Casanova JL (2011) Inborn errors of anti-viral interferon immunity in humans. Curr Opin Virol 1: 487–496. 10.1016/j.coviro.2011.10.016 22347990 PMC3280408

[25] Rosato PC, Leib DA (2015) Neuronal interferon signaling is required for protection against Herpes Simplex Virus replication and pathogenesis. PLoS Pathog 11: e1005028 10.1371/journal.ppat.1005028 26153886 PMC4495997

[26] Aricò E, Monque DM, D'Agostino G, Moschella F, Venditti M, et al (2011) MHV-68 producing mIFNα1 is severely attenuated in vivo and effectively protects mice against challenge with wt MHV-68. Vaccine 29: 3935–3944. 10.1016/j.vaccine.2011.03.092 21481326

[27] Frederico B, Milho R, May JS, Gillet L, Stevenson PG (2012) Myeloid infection links epithelial and B cell tropisms of Murid Herpesvirus-4. PLoS Pathog 8: e1002935 10.1371/journal.ppat.1002935 23028329 PMC3447751

[28] Turco J, Winkler HH (1991) Comparison of properties of virulent, avirulent, and interferon-resistant Rickettsia prowazekii strains. Infect Immun 59: 1647–1655. 1708354 10.1128/iai.59.5.1647-1655.1991 PMC257897

[29] Lawler C, Milho R, May JS, Stevenson PG (2015) Rhadinovirus host entry by co-operative infection. PLoS Pathog 11: e1004761 10.1371/journal.ppat.1004761 25790477 PMC4366105

[30] Guerrero-Plata A, Baron S, Poast JS, Adegboyega PA, Casola A, Garofalo RP (2005) Activity and regulation of alpha interferon in respiratory syncytial virus and human metapneumovirus experimental infections. J Virol 79: 10190–10199. 16051812 10.1128/JVI.79.16.10190-10199.2005 PMC1182647

[31] Weslow-Schmidt JL, Jewell NA, Mertz SE, Simas JP, Durbin JE, et al (2007) Type I interferon inhibition and dendritic cell activation during gammaherpesvirus respiratory infection. J Virol 81: 9778–9789. 17626106 10.1128/JVI.00360-07 PMC2045419

[32] Sarawar SR, Cardin RD, Brooks JW, Mehrpooya M, Hamilton-Easton AM, Mo XY, Doherty PC (1997) Gamma interferon is not essential for recovery from acute infection with murine gammaherpesvirus 68. J Virol 71: 3916–3921. 9094668 10.1128/jvi.71.5.3916-3921.1997 PMC191543

[33] Sarawar SR, Brooks JW, Cardin RD, Mehrpooya M, Doherty PC (1998) Pathogenesis of murine gammaherpesvirus-68 infection in interleukin-6-deficient mice. Virology 249: 359–366. 9791027 10.1006/viro.1998.9309

[34] Egli A, Santer DM, O'Shea D, Tyrrell DL, Houghton M (2014) The impact of the interferon-lambda family on the innate and adaptive immune response to viral infections. Emerg Microbes Infect 3: e51 10.1038/emi.2014.51 26038748 PMC4126180

[35] Kühn R, Schwenk F, Aguet M, Rajewsky K (1995) Inducible gene targeting in mice. Science 269: 1427–1429. 7660125 10.1126/science.7660125

[36] Staeheli P, Danielson P, Haller O, Sutcliffe JG (1986) Transcriptional activation of the mouse Mx gene by type I interferon. Mol Cell Biol 6: 4770–4774. 3796617 10.1128/mcb.6.12.4770-4774.1986 PMC367267

[37] Hug H., Costas M., Staeheli P., Aebi M., and Weissmann C. (1988). Organization of the murine Mx gene and characterization of its interferon- and virus-inducible promoter. Mol. Cell. Biol. 8, 3065–3079. 2974922 10.1128/mcb.8.8.3065-3079.1988 PMC363533

[38] Simon A, Fäh J, Haller O, Staeheli P (1991) Interferon-regulated Mx genes are not responsive to interleukin-1, tumor necrosis factor, and other cytokines. J Virol 65: 968–971. 1702845 10.1128/jvi.65.2.968-971.1991 PMC239840

[39] Pletneva LM, Haller O, Porter DD, Prince GA, Blanco JC (2006) Interferon-inducible Mx gene expression in cotton rats: cloning, characterization, and expression during influenza viral infection. J Interferon Cytokine Res 26: 914–921. 17238834 10.1089/jir.2006.26.914

[40] van Berkel V, Preiter K, Virgin HW, Speck SH (1999) Identification and initial characterization of the murine gammaherpesvirus 68 gene M3, encoding an abundantly secreted protein. J Virol 73: 4524–4529. 10196360 10.1128/jvi.73.5.4524-4529.1999 PMC104349

[41] Simas JP, Swann D, Bowden R, Efstathiou S (1999) Analysis of murine gammaherpesvirus-68 transcription during lytic and latent infection. J Gen Virol 80: 75–82. 9934687 10.1099/0022-1317-80-1-75

[42] Milho R, Smith CM, Marques S, Alenquer M, May JS, et al (2009) In vivo imaging of murid herpesvirus-4 infection. J Gen Virol 90: 21–32. 10.1099/vir.0.006569-0 19088269 PMC2885022

[43] Marques S, Efstathiou S, Smith KG, Haury M, Simas JP (2003) Selective gene expression of latent murine gammaherpesvirus 68 in B lymphocytes. J Virol 77: 7308–7318. 12805429 10.1128/JVI.77.13.7308-7318.2003 PMC164786

[44] Hertzog PJ, Hwang SY, Kola I (1994) Role of interferons in the regulation of cell proliferation, differentiation, and development. Mol Reprod Dev 39: 226–232. 7530016 10.1002/mrd.1080390216

[45] Weck KE, Dal Canto AJ, Gould JD, O'Guin AK, Roth KA, et al (1997) Murine gamma-herpesvirus 68 causes severe large-vessel arteritis in mice lacking interferon-gamma responsiveness: a new model for virus-induced vascular disease. Nat Med 3: 1346–1353. 9396604 10.1038/nm1297-1346

[46] Chao B, Frederico B, Stevenson PG (2015) B cell-independent lymphoid tissue infection by a B cell-tropic rhadinovirus. J Gen Virol 96: 2788–2793. 10.1099/vir.0.000188 25986632

[47] May JS, Coleman HM, Smillie B, Efstathiou S, Stevenson PG (2004) Forced lytic replication impairs host colonization by a latency-deficient mutant of murine gammaherpesvirus-68. J Gen Virol 85: 137–146. 14718628 10.1099/vir.0.19599-0

[48] Marshall EE, Geballe AP (2009) Multifaceted evasion of the interferon response by cytomegalovirus. J Interferon Cytokine Res 29: 609–619. 10.1089/jir.2009.0064 19708810 PMC2743745

[49] Hsu KM, Pratt JR, Akers WJ, Achilefu SI, Yokoyama WM (2009) Murine cytomegalovirus displays selective infection of cells within hours after systemic administration. J Gen Virol 90: 33–43. 10.1099/vir.0.006668-0 19088270 PMC2762738

[50] Dağ F, Dölken L, Holzki J, Drabig A, Weingärtner A, et al (2014) Reversible silencing of cytomegalovirus genomes by type I interferon governs virus latency. PLoS Pathog 10: e1003962 10.1371/journal.ppat.1003962 24586165 PMC3930589

[51] Hwang SY, Hertzog PJ, Holland KA, Sumarsono SH, Tymms MJ, et al (1995) A null mutation in the gene encoding a type I interferon receptor component eliminates antiproliferative and antiviral responses to interferons alpha and beta and alters macrophage responses. Proc Natl Acad Sci USA 92: 11284–11288. 7479980 10.1073/pnas.92.24.11284 PMC40616

[52] Everett RD, Chelbi-Alix MK (2007) PML and PML nuclear bodies: implications in antiviral defence. Biochimie 89: 819–830. 17343971 10.1016/j.biochi.2007.01.004

[53] Gaspar M, Gill MB, Lösing JB, May JS, Stevenson PG (2008) Multiple functions for ORF75c in murid herpesvirus-4 infection. PLoS One 3: e2781 10.1371/journal.pone.0002781 18648660 PMC2464709

[54] Ling PD, Tan J, Sewatanon J, Peng R (2008) Murine gammaherpesvirus 68 open reading frame 75c tegument protein induces the degradation of PML and is essential for production of infectious virus. J Virol 82: 8000–8012. 10.1128/JVI.02752-07 18508901 PMC2519593

[55] Full F, Jungnickl D, Reuter N, Bogner E, Brulois K, et al (2014) Kaposi's sarcoma associated herpesvirus tegument protein ORF75 is essential for viral lytic replication and plays a critical role in the antagonization of ND10-instituted intrinsic immunity. PLoS Pathog 10: e1003863 10.1371/journal.ppat.1003863 24453968 PMC3894210

[56] Tsai K, Thikmyanova N, Wojcechowskyj JA, Delecluse HJ, Lieberman PM (2011) EBV tegument protein BNRF1 disrupts DAXX-ATRX to activate viral early gene transcription. PLoS Pathog 7: e1002376 10.1371/journal.ppat.1002376 22102817 PMC3213115

[57] Stevenson PG, May JS, Smith XG, Marques S, Adler H, et al (2002) K3-mediated evasion of CD8(+) T cells aids amplification of a latent gamma-herpesvirus. Nat Immunol 3: 733–740. 12101398 10.1038/ni818

[58] Adler H, Messerle M, Wagner M, Koszinowski UH (2000) Cloning and mutagenesis of the murine gammaherpesvirus 68 genome as an infectious bacterial artificial chromosome. J Virol 74: 6964–6974. 10888635 10.1128/jvi.74.15.6964-6974.2000 PMC112213

[59] May JS, Stevenson PG (2010) Vaccination with murid herpesvirus-4 glycoprotein B reduces viral lytic replication but does not induce detectable virion neutralization. J Gen Virol 91: 2542–2552. 10.1099/vir.0.023085-0 20519454 PMC3052599

[60] Farrell HE, Davis-Poynter N, Bruce K, Lawler C, Dolken L, et al (2015). Lymph Node Macrophages Restrict Murine Cytomegalovirus Dissemination. J Virol 89: 7147–7158. 10.1128/JVI.00480-15 25926638 PMC4473555

[61] de Lima BD, May JS, Stevenson PG (2004) Murine gammaherpesvirus 68 lacking gp150 shows defective virion release but establishes normal latency in vivo. J Virol 78: 5103–5112. 15113892 10.1128/JVI.78.10.5103-5112.2004 PMC400354

