# Supplementary figures and images for: Type I Interferons Direct Gammaherpesvirus Host Colonization
Source: PLoS Pathog. 2016 May 25;12(5):e1005654. doi: 10.1371/journal.ppat.1005654 (PMC4880296; doi:10.1371/journal.ppat.1005654)

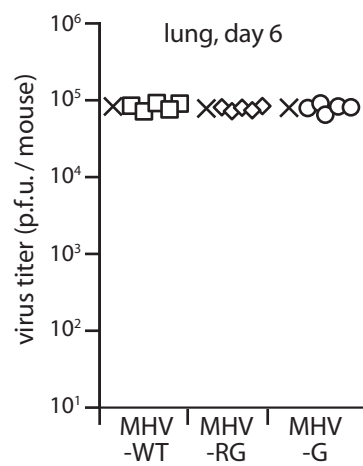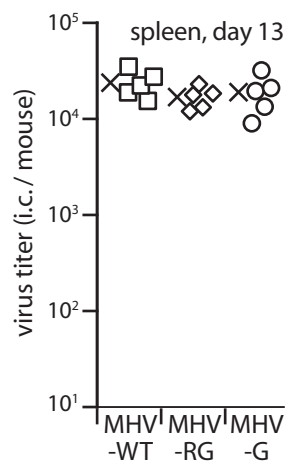

Supplement: S1 Fig — C57BL/6 mice were infected in lungs (104 p.f.u. i.n. in 30μl under anesthesia) with wild-type MuHV-4 (MHV-WT), colour switching MuHV-4 (MHV-RG), or the same virus pre-switched in vitro by passage through cre+ NIH-3T3 cells (MHV-G). Lungs were titered for infectious virus after 6 days by plaque assay. Spleens were titered for total recoverable virus after 13 days by infectious center assay. Crosses show means, other symbols show individual mice. No significant difference was seen between MHV-WT, MHV-RG and MHV-G. (PDF) [file ppat.1005654.s001.pdf]

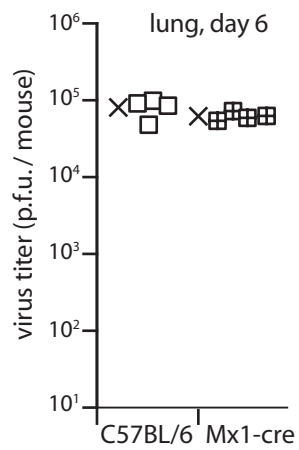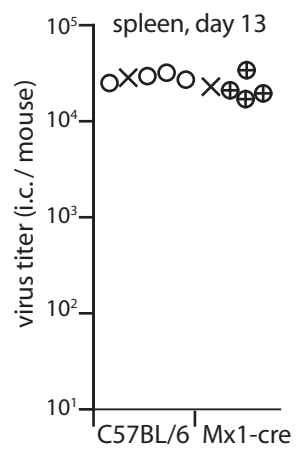

Supplement: S2 Fig — Mx1-cre mice were bred as heterozygote x non-transgenic. Mx1-cre+ heterozygote progeny (Mx1-cre) and non-transgenic littermates (C57BL/6) were infected i.n. when 6–8 weeks old with wild-type MuHV-4 (104 p.f.u. i.n. in 30μl under isoflurane anesthesia). Infectious virus in lungs was titered after 6 days by plaque assay, and lytic plus latent virus in spleens was titered after 13 days by infectious center assay. Crosses show means; other symbols show individual mice. No significant difference was observed between Mx1-cre+ and C57BL/6 mice. (PDF) [file ppat.1005654.s002.pdf]

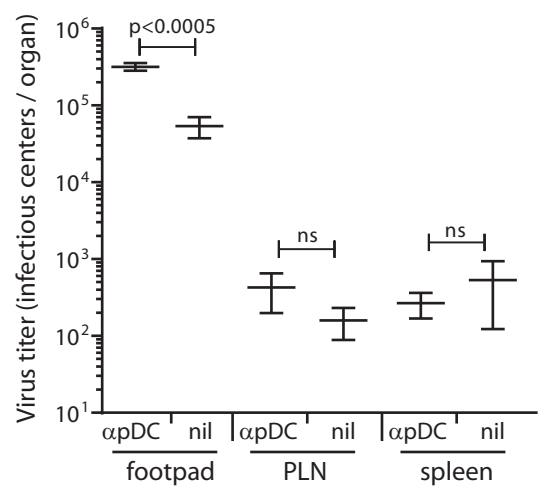

Supplement: S3 Fig — Mice were gives anti-pDC mAb (3x400μg, mAb BX444, anti-CD317/BST2/PDCA-1, Bio X Cell) or not i.p., then MuHV-4 into footpads (105 p.f.u.). 3 days later footpads, popliteal lymph nodes (PLN) and spleens were titered for virus by infectious center assay. Bars show mean ± SEM for 3–6 mice. Virus titers were significantly reduced in footpads by Student’s unpaired 2 tailed t test, but not in PLN or spleens (ns = not significant, p>0.05). (PDF) [file ppat.1005654.s003.pdf]

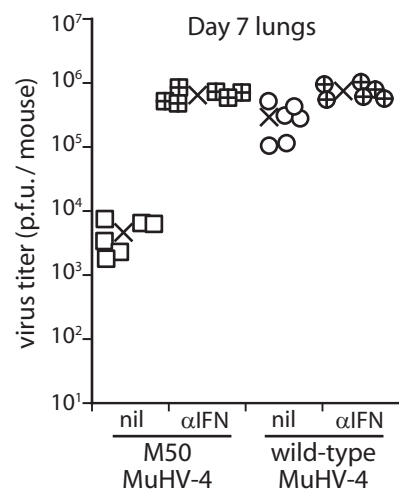

Supplement: S4 Fig — Mice were given anti-IFNAR blocking mAb (100μg i.p. every other day, αIFN) or not (nil) then wild-type (WT) or M50 MuHV-4 i.n. (105 p.f.u.). M50 MuHV-4 has the proximal 416bp of the Murine cytomegalovirus IE1 promoter inserted in its ORF50 exon1 5’ untranslated region. ORF50 encodes the MuHV-4 lytic switch protein. M50 MuHV-4 shows increased ORF50 transcription and an incapacity to remain latent (May JS, Coleman HM, Smillie B, Efstathiou S, Stevenson PG (2004) Forced lytic replication impairs host colonization by a latency-deficient mutant of murine gammaherpesvirus-68. J Gen Virol 85: 137–146). At 7 days after infection, lungs were titered for infectious virus by plaque assay. Crosses show means, other symbols show individual mice. Without aIFN mAb M50 titers were significantly less than wild-type (p<0.001 by Student’s unpaired 2-tailed test); with IFNAR blockade M50 and WT titers were not significantly different. (PDF) [file ppat.1005654.s004.pdf]

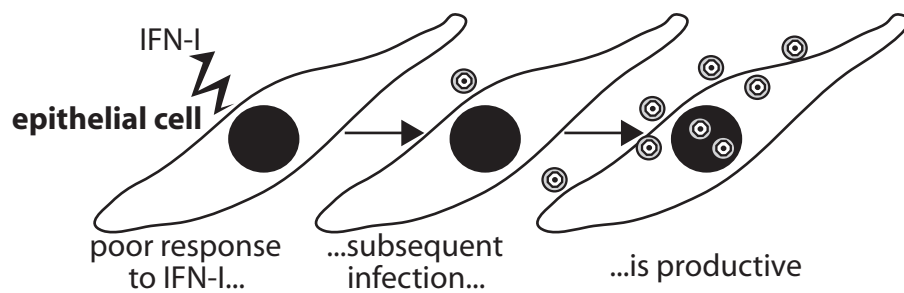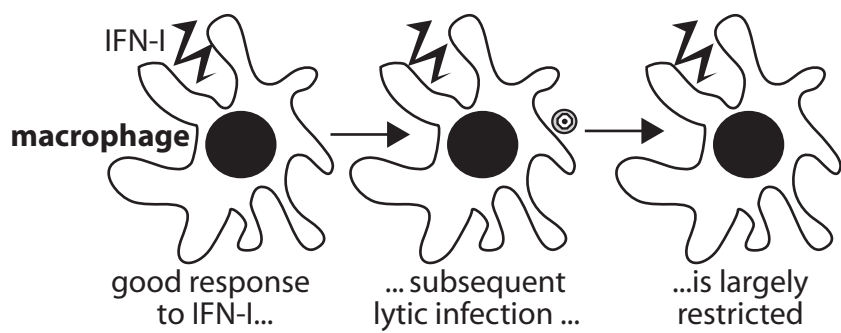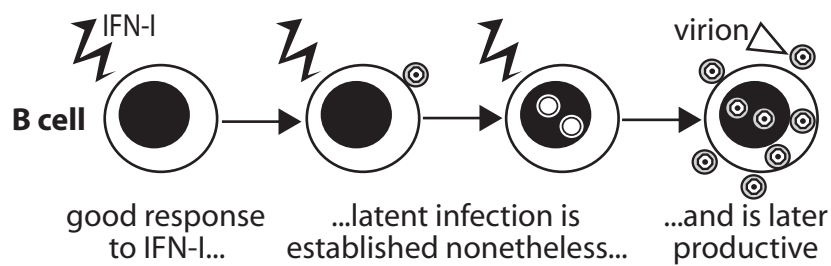

Supplement: S5 Fig — Type 1 alveolar epithelial cells made no detectable Mx1 response to MuHV-4 infection or to p(I:C), and IFN-I induction had little effect on viral replication in the lungs, where these cells are abundantly infected. Thus, their interaction was dominated by poor responsiveness to IFN-I. Macrophages contrastingly showed viral fluorochrome switching but propagated switched virions poorly, and IFN-I blockade increased massively the extent of their infection. Thus, in macrophages IFN-I was protective. B cells were different again. They showed abundant viral fluorochrome switching and switched virion production. IFN-I blockade had little effect on infection, but viral evasion gene disruption caused marked attenuation, indicating that B cell infection is normally dominated by IFN-I evasion. This implies that virions can enter IFN-I-responding B cells and establish a latent infection that is stably maintained and can reactivate, presumably when IFN-I signalling has diminished. (PDF) [file ppat.1005654.s005.pdf]
